# Effect of health belief model on flood-risk educational approach among elementary school children in Malaysia

**DOI:** 10.4102/jamba.v13i1.1102

**Published:** 2021-07-30

**Authors:** Ezza S. Azmi, Vivien How, Haliza Abdul Rahman

**Affiliations:** 1 Department of Environmental and Occupational Health, Faculty of Medicine and Health Sciences, Universiti Putra Malaysia, Serdang, Malaysia; 2 Institute for Social Science Studies, Universiti Putra Malaysia, Serdang, Malaysia

**Keywords:** health belief model, flood-risk reduction, knowledge transfer, school children

## Abstract

Worsening climatic conditions can subsequently lead to the frequent occurrence of unpredictable natural disasters. The early-life educational approach is one of the non-structural mitigations in disaster management, which are the most effective efforts to promote early-life disaster awareness and enhance the knowledge transfer in disaster risk education. By using the health belief model (HBM), this study aims to examine the effectiveness of HBM on the flood-risk reduction (FRR) educational intervention by looking into the perceived susceptibility, severity, benefit and self-efficacy among elementary school children in Malaysia. This study utilised the one-group pre-test–post-test design by recruiting 224 elementary school children in the pre-FRR educational intervention programme, and 205 who undertook a post-intervention programme a month later. This study showed that the FRR educational intervention significantly improved (*p* < 0.001) the overall HBM components during the post-intervention, particularly in: (1) FRR knowledge, (2) perceived susceptibility, (3) perceived severity and (4) perceived benefits. The one-way analysis of covariance test showed that knowledge transfer intervention is effective to improve all the HBM components that include (1) FRR knowledge, *F*(38,127) = 2.517; (2) perceived susceptibility, *F*(6,191) = 6.957; (3) perceived severity, *F*(20,163) = 2.944; (4) perceived benefits, *F*(25,153) = 2.342 and (5) self-efficacy, *F*(7,189) = 12.526. The impact of integrating HBM into knowledge transfer intervention was seen to be effective and provide a positive knowledge enhancement among learners. Therefore, it is crucial to implement a consistent and sustainable educational intervention to harness formal education for community resilience at an early age.

## Introduction

Malaysia is strategically located outside the ‘Pacific Rim of Fire’ and the typhoon pathway, which then protects the country from severe natural disasters such as earthquakes, volcanic eruptions and typhoons (Educational Research and Innovation Office [ERIO] 2014; How et al. 2015; National Institute of Disaster Management [NIDM] 2014). Despite that, the country had experienced the hydrological type of disasters such as recent data on natural disaster show that flood (62%) is the disaster that frequently strikes most places in Malaysia followed by storm (13%) and landslide (8%) (International Disaster Database 2015; NIDM 2014). Climate change is responsible for increasing sea levels and rainfall, which lead to increase flood risks. In addition, poor drainage system management in most of the cities exposes Malaysia to flood risks.

Although disasters caused by extreme weather are inevitable because of the changing climate pattern (Loo, Billa & Singh 2015; Rahman & Suppian 2018), the impact can be minimised by adapting to the changing climate and implementing a sustainable mitigation measure. In Malaysia, most efforts have been focused on the control of flood impact via structural mitigation measures by enhancing climate-resistant infrastructure and facilities (Department of Drainage and Irrigation [DID] 2016). Non-structural measures, on the other hand, are a set of mitigation and/or adaptation measures that aim to reduce the flood-induced damage through an integrated risk management approach, which include: education intervention, spatial planning, flood action plans, emergency response and so on (DID 2016; UN Office for Disaster Risk Reduction [UNDRR] 2020). The focus for this study is on educational intervention as one of the diverse non-structural measures to mitigate flood-risk damage.

Past study (Jani, Nasir & Zawawi 2015) has shown that flood preparedness and awareness among Malaysians are relatively low as compared to other disaster-prone countries in Asia such as Japan or Taiwan. This may be attributed to the fact that most Malaysians have yet to experience a catastrophe that has caused immense property damage and mortality losses at once. In other words, the disaster resilience level remains poor among the community (Dorasamy 2010), especially the children. Children are vulnerable to flood hazards as they are physically small in size, lack strength and also emotionally dependant on adults during a catastrophe (Morrow 1999; Twigg 2015). Given this, an effective disaster educational intervention has been seen as a crucial measure at an early age to enhance early disaster preparedness and adaptation measures for a sustainable resilience community in the long run (McLeod 2013).

The health belief model (HBM) is an educational framework widely utilised by health educators since the 1950s to intervene in health-related behavioural changes. The main structure of HBM contains elements (Green, Murphy & Gryboski 2020; Shojaei et al. 2016), such as:

- perceived sustainability (individual perception of getting the diseases’ risk or hazards from an unwanted event)
- perceived severity (individual awareness of the seriousness of getting diseases or affected by an unwanted event)
- perceived benefit (individual perception of the effectiveness of the actions that are available to reduce the potential health risks)
- perceived barrier (individual belief about the potential effect of adopting the promoted action)
- cue to action (an action that triggers the decision-making process and determines the readiness).

**FIGURE 1:**
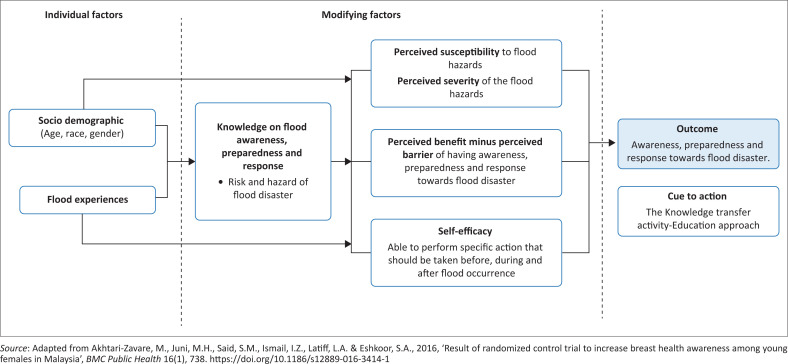
Theoretical framework of integrating health belief model into flood-risk educational intervention.

Past studies have shown that a strategised educational approach would positively change one’s health belief (Shojaei et al. 2016), which is supported by a breast health awareness intervention study performed by Akhtari-Zavare et al. (2016), who indicated that the HBM educational approach changes the habit of self-breast examination effectively among study respondents. Considering the conceptual of HBM, this study integrates the HBM framework into flood-risk reduction (FRR) educational intervention (see Figure 1) by presuming that a sustainable FRR could be achieved by inculcating knowledge transfer at an early age.

## Methodology

This is a one-group pre-test–post-test quasi-experimental study where FRR educational intervention was performed to examine the effectiveness of flood-risk knowledge transfer level among elementary school children by integrating the HBM framework.

A total of 224 elementary school children were recruited in the pre-FRR educational intervention programme, and 205 undertook a post-intervention programme a month later. Their schools were selected randomly based on the list of schools obtained from the District Education Office. A probability sampling method was used to randomly recruit school children who fulfil the inclusive criteria from the selected schools. These criteria include students aged between 9 and 10 years old and have signed the consent letter from their parents or guardians before participating in this intervention programme.

### Flood-risk education intervention programme

This intervention was mainly developed based on the working target of the Sendai Framework (2015–2030) (UN Office for Disaster Risk Reduction [UNDRR] 2019) aiming to strengthen the protection capacities in risk-prone environments by preventing new and minimising current disaster risk through the implementation of an integrated and inclusive educational programme. Therefore, the FRR teaching module for this intervention programme consists of five HBM components as summarised in Table 1 to cover the flood-risk learning module and its learning outcome that integrated into the HBM framework.

**TABLE 1:** Learning module for the flood-risk education intervention programme integrated into health belief model framework.

Number	Learning module	Learning outcome (LO)	HBM component
1	General flood knowledge	Learner to expose the fundamental knowledge of floods’ science and its relevancy to climate condition	FRR knowledge
2	Flood warning	Learner to recognise the flood warning system during the disaster response phase	Perceive susceptibility and perceived severity
3	Assembling emergency kit	Learner to assemble the flood emergency kit during disaster response and recovery phase	Perceived benefit
4	Readiness or response to flood	Learner to demonstrate their readiness to respond to a flood disaster with the knowledge they learn from learning modules 1–3.	Self-efficacy

HBM, health belief model; FRR, flood-risk reduction.

The effectiveness of this flood-risk education intervention programme was evaluated based on a one-group pre-test–post-test study design, that is, pre- and post-intervention at the 1-month interval. Pre-test intervention was evaluated by using the validated questionnaire to determine the fundamental knowledge of flood hazards and risk throughout different disaster phases among the study population. Based on the learning module, the questionnaire or quiz set is divided into five parts, which are: (1) general flood knowledge, (2) flood warning, (3) assembling emergency kit, (4) readiness or response to flood and (5) socio-demographical information.

**FIGURE 2:**
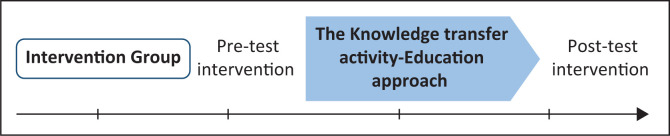
The activity flow of flood-risk education intervention programme.

Following the pre-test assessment session, the researcher rendered the learning module with an interactive learning session to school children. The knowledge transfer intervention activity was conducted by the researcher to fulfil the learning outcome for each learning module, and the whole session was completed in 1h – 2 h on the same day. One-month following the pre-intervention programme, the post-test assessment was conducted by disseminating the same set of questionnaires as in the pre-test assessment to re-evaluate the changes of knowledge levels on FRR. Figure 2 summarises the activity flow of the flood-risk education intervention programme.

### Analysis of data

Statistical Package for Social Science (SPSS) version 22.0 was performed to analyse the data obtained from the one-group pre-test–post-test study. Socio-demographical data were presented in percentage, while paired sample *t*-test was used to compare the baseline knowledge before and after the intervention programme. By considering flood experience as a covariate, the one-way analysis of covariance (ANCOVA) was used to identify the effectiveness of the knowledge transfer intervention based on the HRM framework

## Results

### Socio-demographical information

A total of 224 elementary school children were recruited in the pre-FRR educational programme and 205 undertook a post-intervention programme a month later. Therefore, the socio-demographical information of the 205 elementary school students was summarised as shown in Table 2. This study includes 53.7% of female students and 66.3% of male students at the ages of 9–10 years old. Most of the students are of Malay ethnicity (98%), followed by Indian (1.5%) and other ethnic minorities (0.5%). Overall, the majority (74.1%) of school children have never experienced a flood before, while only 25.9% of them claimed that they have experienced flood.

**TABLE 2:** The socio-demographic information of the school children (*N* = 205).

Respondents	Variable	*n*	%
Gender	Male	95	46.3
	Female	110	53.7
Age	9 years old	69	33.7
	10 years old	136	66.3
Origin	Malay people	201	98.0
	Chinese people	0	0.0
	Indian people	3	1.5
	Others	1	0.5
Flood experiences	Yes	53	25.9
	No	152	74.1

### Comparison pre-test–post-test intervention

Based on the HRM framework, the mean scores of the FRR knowledge level have shown significant differences (*p* < 0.001) before and after the knowledge transfer intervention activity, except for the self-efficacy (*p* = 0.206). Nevertheless, all the HBM components presented an increase of scores after the intervention programme. For instance, the total score for the FRR knowledge level revealed significant differences (*p* < 0.001) with a mean difference of -5.259 (-6.717, -3.800); the mean difference of the perceived susceptibility is -0.498 (-0.700, -0.295); the mean difference of the perceived severity part is -1.263 (-1.948, -0.579); and the mean difference in the perceived benefits part is -3.366 (-4.248, -2.484), all of which showed a significant difference at the pre-test–post-test activity.

### The effectiveness of the knowledge transfer intervention with flood experience as co-variate

One-way analysis of covariance was used to identify the effectiveness of the knowledge transfer intervention to improve FRR knowledge level and the HBM components among the elementary school children. The test was performed after statistically controlling for the effects of flood experience that is considered as a covariate. The result shows that the knowledge transfer intervention is effective to improve all the HBM components, except for ‘self-efficacy’. Other shows the significant difference and improvement on the HBM component, such that FRR knowledge, *F*(38,127) = 2.517; perceived susceptibility, *F*(6,191) = 6.957; perceived severity, *F*(20,163) = 2.944; and perceived benefits with *F*(25,153) = 2.342. The data were tabulated in Table 4.

**TABLE 3:** The pre-post comparison of the flood-risk reduction knowledge level and health belief model component at baseline and after a month of knowledge transfer intervention (*N* = 205).

Part	Before		After		Mean difference	95% CI	*t*	*p*-value
	Mean	SD	Mean	SD				
Knowledge	98.76	9.492	104.02	9.294	−5.259	−6.717, −3.800	−7.110	< 0.001*
(total marks = 114 marks)								
Susceptibility	8.76	1.382	9.25	1.238	−0.498	−0.700, −0.295	−4.855	≤ 0.001*
(total marks = 10 marks)								
Severity	38.46	4.241	39.72	4.315	−1.263	−1.948, −0.579	−3.638	≤ 0.001*
(total marks = 44 marks)								
Benefits	40.20	5.535	43.56	4.858	−3.366	−4.248, −2.484	−7.524	< 0.001*
(total marks = 48 marks)								
Self-efficacy	11.35	1.380	11.48	1.235	−0.132	−0.337, 0.073	−1.268	0.206
(total marks = 12 marks)								

SD, standard deviation; CI, confidence interval.

* , *p*-value significant at *p* < 0.001.

**TABLE 4:** The effectiveness of the knowledge transfer intervention with flood experience as covariate.

Factors†	Mean square	*F*-statistics	*df*	*p*	*R*^2^
Knowledge (total marks = 114 marks)	169.802	2.517	38, 127	< 0.001*	0.219
Susceptibility (total marks = 10 marks)	9.114	6.957	6, 191	< 0.001*	0.146
Severity (total marks = 44 marks)	46.192	2.944	20, 163	< 0.001*	0.158
Benefits (total marks = 48 marks)	47.601	2.342	25, 153	0.001*	0.139
Self-efficacy (total marks = 12 marks)	13.637	12.526	7, 189	0.067	0.286

† , One-way analysis of covariance (ANCOVA).

* , *p*-value significant at *p* ≤ 0.05.

## Discussion

Health belief model showed to be an effective approach to enhance the understanding of an individual, improve motivation and provide resources to explain social and cultural beliefs and attitudes towards disaster-related knowledge (Inal, Altintas & Dogan 2018; Inal & Dogan 2018). Therefore, it is deemed important to understand the background of one’s disaster-related knowledge, which helps to improve attitudes and practices towards disaster preparedness and response gradually.

Based on the HBM framework that we utilised in this study, the result showed that there is an improvement in the FRR knowledge level after introducing the knowledge transfer intervention programme to school children. The finding of this study is also supported by previous work conducted by Akhtari-Zavare et al. (2016), who suggested that HBM-based education-based intervention significantly improved breast cancer knowledge among the study population. Another study conducted by Cheraghi et al. (2014) also recommended that utilising the HBM-based knowledge transfer approach has enhanced the mother’s knowledge, attitude and practices regarding injury prevention among children aged below 5 years old.

Apart from the fact that FRR knowledge demonstrates the positive changes in knowledge, the positive changes in ‘perceived susceptibility’ and ‘perceived severity’ among school children are worth discussing in this context. This is important for an individual to account for their ‘perceived susceptibility’ and ‘perceived severity’, respectively, as it would influence how they prepared themselves while reacting to the unfortunate situation. According to Qasim et al. (2015) in a study of the risk perception of the people in the flood-prone areas in Pakistan, it was found that the risk perception was strongly correlated with the flood risks and subsequently influenced by a disaster risk reduction activity. The knowledge of public perception is thought to be important for good flood management. Therefore, using HBM as the basis for FRR knowledge transfer among the school children has shown an increase in their perception of flood risk and the severity of the impacts because of the occurrence of the unexpected event.

On the other hand, the ‘perceived benefit’ among school children has been shown to increase significantly in this study. This is consistent with a past study by Jassempour et al. (2014), which indicated the effectiveness of implementing an educational intervention to enhance the perceived benefit of having a disaster survival kit. This explained the similar phenomenon found in this study where the school children are more likely to perceive the benefits to enhance their level of knowledge on FRR, as well as the effects of not having proper knowledge on FRR.

Self-efficacy is required in individuals to make a change for better behaviour; however, this study showed no significant difference in self-efficacy among the school children following the knowledge transfer intervention. Past study to determine the effectiveness of the HBM-based educational approach to nutritional knowledge and behaviour has similar findings with this present study. They have found a positive increase in nutritional knowledge, perceived severity, perceived benefits and barriers, except the dietary behaviour between the two compared groups (Shojaei et al. 2016).

Although there is no doubt that knowledge transfer intervention can improve the level of FRR knowledge of the school children, it is insufficient to convert their behaviour into action with a short-term and immediate educational-intervention approach. Self-efficacy, on the other hand, is someone who believes in their ability and motivates them to succeed in a certain situation and able to change their behaviour to be better. In other words, a continuous good self-efficacy is important to influence behaviour modification (Srithongklang et al. 2019). This is where self-efficacy should be in-placed to ensure long-term and sustainable changes in disaster risk education starting from an early age. In other words, the improvement of the knowledge alone as demonstrated from the output of this study seems not enough to motivate the school children to be prepared for the upcoming flood occurrences. A sustainable lifelong learning process is needed not only to improve the knowledge but also to encourage an individual to perform good practices and for them to feel responsible for their safety and health, starting from an early age.

## Conclusion

This study concluded that integrating the HBM framework into FRR educational intervention has promising outcome on the effectiveness of raising the level of knowledge on FRR among the school children and improves the HBM components. Although the self-efficacy in the HBM component was yet to be improved accordingly, consistent and sustainable intervention is required to appropriately create better preparedness and readiness towards flood occurrences, and thus, indirectly create a resilient community towards disaster in the future. Implementation of the integration of FRR in teaching and learning or the school curriculum is also seen to be the best platform as a start to educate the community regarding flood-related knowledge from an early age.
